# Genetic Diversity and Population Differentiation of *Calanthe tsoongiana*, a Rare and Endemic Orchid in China

**DOI:** 10.3390/ijms141020399

**Published:** 2013-10-14

**Authors:** Xin Qian, Cai-xia Wang, Min Tian

**Affiliations:** Research Institution of Subtropical Forestry, Chinese Academy of Forestry, 73, Daqiao Road, Fuyang 311400, Zhejiang, China; E-Mails: qianxin090@163.com (X.Q.); cxwanghn@163.com (C.W.)

**Keywords:** *Calanthe tsoongiana*, genetic diversity, ISSR, conservation, Orchidaceae

## Abstract

*Calanthe tsoongiana* is a rare terrestrial orchid endemic to China, and this species has experienced severe habitat loss and fragmentation. Inter-simple sequence repeat (ISSR) markers were employed to assess the genetic diversity and differentiation of six populations of *C. tsoongiana*. Based on 124 discernible fragments yielded by eleven selected primers, high genetic diversity was revealed at the species level; however, genetic diversity at the population level was relatively low. High-level genetic differentiation among populations was detected based on analysis of molecular variance (AMOVA), indicating potential limited gene flow. No significant relationship was observed between genetic and geographic distances among the sampled populations. These results suggested that restricted gene flow might be due to habitat fragmentation and reduced population size as a result of human activities. Based on the findings, several conservation strategies were proposed for the preservation of this threatened species.

## 1. Introduction

Orchidaceae is one of the largest families of flowering plants, with approximately 25,000 species comprising up to 10% of all angiosperms, including five subfamilies and about 870 genera [1]. More than any other plant family, however, Orchidaceae has a large proportion of threatened species that only survive in specific environments and may be particularly susceptible to habitat fragmentation and deterioration caused by human activities, such as over-collection, due to their ornamental and medicinal value [1]. Therefore, worldwide orchid populations are often small and isolated and have increasingly been a major subject of conservation concern. The orchid genus, *Calanthe* R. Br., is widely distributed in tropical, subtropical and warm temperate regions from Africa to Asia and the Pacific Islands. It is characterized by distichous plicate leaves, basal or lateral inflorescences, pseudobulbous or cane-like stems made up of several internodes, similar petals and sepals, normally spurred lips and possession of eight pollinia [2]. This genus contains approximately 171 species worldwide, 40 of which inhabit China [2]. *Calanthe tsoongiana* T. Tang et F.T. Wang is a rare perennial orchid endemic to China and is mainly distributed in Fujian, Guizhou, Hunan, Jiangxi and Zhejiang provinces [3]. They usually grow in forest margins and rocky slopes at elevations of 300 to 1500 m [3]. The habitat range and population size of *C. tsoongiana* has declined acutely during the last several decades, due to climatic change, agriculture, urbanization, silviculture and mass collection for horticultural use and sale. Many populations recorded previously have disappeared, and current distributions are highly fragmented and isolated. Recently, this species has been listed as a national second class protection species in China [4]. Except for its taxonomic and morphologic characteristics, however, this species has been poorly studied. To date, nothing is known about its within-population or its intra-specific genetic diversity and population structure among the distribution areas.

Comprehensive knowledge on genetic diversity and population structure is essential for species conservation, as it reflects the status and survival potential of populations [5]. The maintenance of genetic diversity is very important for the long-term survival of a species, because loss of genetic variation within populations may significantly decrease adaptability to environmental change and increase extinction risk [6]. Disappearance of an individual population will remove any unique biological traits that it possesses and may ultimately lead to species extinction [6]. George *et al*. [7] suggested that examining the patterns and levels of genetic diversity within and structuring among populations can help in understanding evolutionary mechanisms, such as genetic drift and mutation operating at the population and species level and can serve as an indicator of the extent of gene flow and population divergence. Among various molecular tools for genetic analysis, inter-simple sequence repeat (ISSR) is a powerful tool for conservation genetic study, due to its greater reliability and reproducibility of bands, resolution of a high number of polymorphic fragments and relatively low cost [8–10]. In addition, because no prior knowledge of target sequences is required for ISSR, this marker is especially suited to rare orchids, where few or no molecular genetic studies have been previously conducted [8,11]. Furthermore, ISSR has been used successfully for studying population genetics in several orchid species, including *Piperia yadonii* [7], *Cymbidium goeringii* [11], *Tipularia discolor* [12], *Platanthera aquilonis* [13], *P. dilatata* [13], *P. huronensis* [13], *Gastrodia elata* [14] and *Dendrobium fimbriatum* [15]. Consequently, the aim of the present study on the genetic diversity and structure of *C. tsoongiana* was to contribute to the pool of background genetic information and to suggest conservation schemes from a genetic perspective.

## 2. Results

### 2.1. Genetic Diversity of *C. tsoongiana*

In total, 104 individuals from six *C. tsoongiana* populations generated 124 clear and discernible bands using eleven selected ISSR primers, of which 120 (96.8%) were polymorphic (Table 1). This indicated that the ISSR markers measured sufficient polymorphism for DNA typing in the population genetic study of *C. tsoongiana*. The number of fragments yielded per primer varied from eight to 14, with an average of 11.3. The size of the amplified products ranged from 100 to 2000 bp. Genetic diversity was relatively low at the population level (percentage of polymorphic loci, *PPL* = 50%; Nei’s gene diversity, *H* = 0.183; Shannon’s Information index, *I* = 0.271; observed number of alleles, *N*_a_ = 1.500; effective number of alleles, *N*_e_ = 1.318) and shifted variably across different sites. The highest and lowest levels of genetic diversity were found in SZ (*PPL* = 72.6%; *H* = 0.284; *I* = 0.415) and WN (*PPL* = 30.7%; *H* = 0.119; *I* = 0.174), respectively. However, the natural populations of *C. tsoongiana* had high levels of genetic diversity at the species level (*PPL* = 96.8%; *H* = 0.398; *I* = 0.576; *N*_a_ = 1.968; *N*_e_ = 1.720).

### 2.2. Genetic Differentiation and Gene Flow in *C. tsoongiana*

The total genetic diversity (*H*_t_) of the species was 0.406, and the genetic diversity within populations (*H*_s_) was 0.183. The coefficient of genetic differentiation among populations (*G*_st_) was about 0.55, indicating that 55% of the genetic variation was among the populations and 45% was within the populations. The analysis of molecular variance (AMOVA) result (Φ_pt_ = 0.522) further revealed that a large proportion of genetic differentiation was partitioned among populations, and the differences among populations were highly significant (*p* = 0.001) (Table 2) [16]. These findings were consistent with the relatively low gene flow value (*N*_m_ = 0.408) obtained among populations.

### 2.3. Genetic Relationships among *C. tsoongiana* Populations

**Figure 1:**
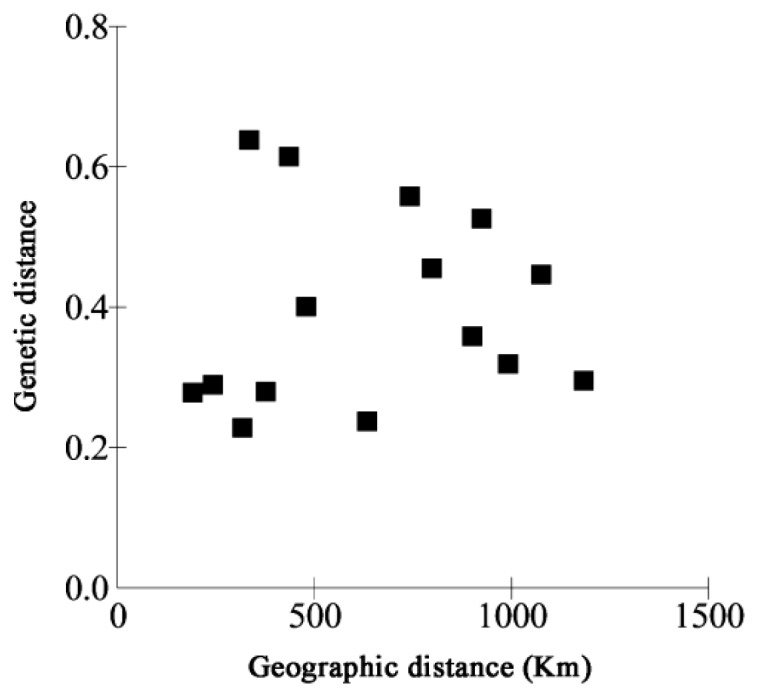
Relationship between genetic and geographic distance in the populations of *C. tsoongiana*.

**Figure 2:**
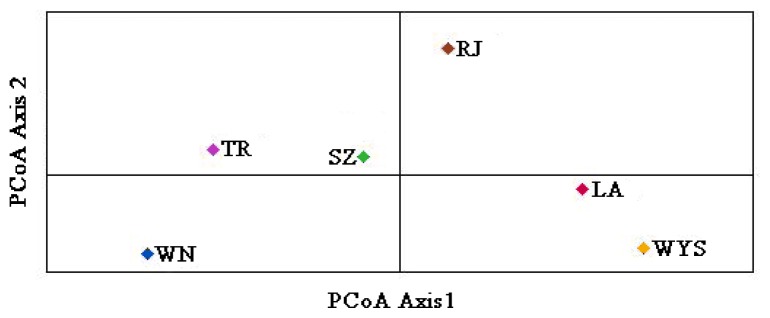
The principal coordinate analysis (PCoA) plot of six populations of *C. tsoongiana* based on the two principal axes (first axis = 43.94%, second axis = 21.14%).

**Figure 3:**
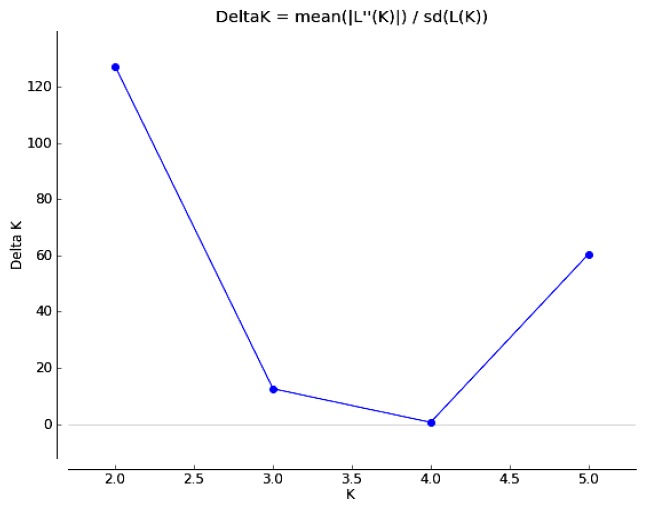
Results of the Bayesian assignment analysis using the Structure Harvester.

**Figure 4:**
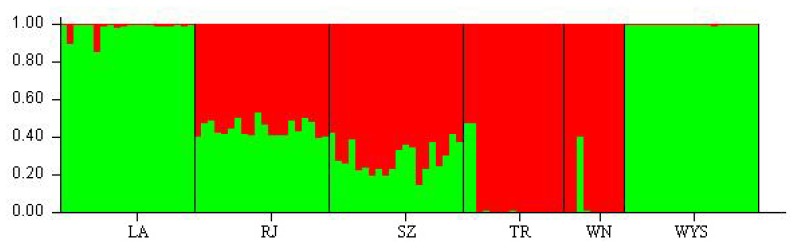
Population structure of six populations of *C. tsoongiana* prepared using the STRUCTURE program (Pritchard Lab, Stanford University, CA, USA).

The Nei’s genetic distance among the *C. tsoongiana* populations ranged from 0.229 (between LA and WYS) to 0.638 (between WN and WYS) (Table 3). The Mantel test indicated that there was no significant correlation between genetic distance and geographic distance (*r* = 0.088, *p* = 0.306) (Figure 1). The UPGMA dendrogram showed general separation of the six populations, but a few individuals from four populations (RJ, JR, SZ and TR) were cluster mixed (not shown). All 104 plants were clustered into two major groups, three populations (LA, WYS and RJ) and two individuals from SZ and two from TR formed the first group, while the second group included the remaining three populations (SZ, TR and WN). Two-dimensional principal coordinate analysis (PCoA) of the six populations accounted for 43.94% (first axis) and 21.14% (second axis) of total variance, respectively (Figure 2). The first axis separated the six populations into two major groups, which accorded with cluster analysis, and the LA and WYS populations occupied a similar position along the second axis. There was a clear peak in the value of Δ*K* (127.2) at *K* = 2 (Figure 3). Therefore *K* = 2 best fit the data. The proportions of all individuals were assigned into two clusters (Figure 4), but the RJ and SZ populations displayed some degree of mixed ancestry, which was analogous to the result of UPGMA dendrogram.

## 3. Discussion

### 3.1. Genetic Diversity

To the best of our knowledge, only a few reports exist on the conservation genetics of the *Calanthe* genus. Chung *et al*. [17] studied the levels and distribution of genetic diversity in *Calanthe discolor*, *C. reflexa* and *C. sieboldii* using allozyme technology and found that genetic variation varied at the species and population level. Kim *et al*. [2] found high levels of polymorphism within three *Calanthe* taxa, including *C. sieboldii*, *C. discolor* and *C. bicolor*. The results of the present study revealed that the genetic diversity of *C. tsoongiana* was low at the population level, but high at the species level. Similar outcomes have been found in several other orchid species based on dominant DNA markers, for example, *Gastrodia elata* [14], *Piperia yadonii* [7] and *Cymbidium goeringii* [11] by inter simple sequence repeat (ISSR), *Dendrobium officinale* [18] and *Liparis japonica* [19] by amplified fragment length polymorphism (AFLP) and *Dendrobium loddigesii* by sequence related amplified polymorphism (SRAP) [20].

Generally, widespread species tend to possess higher genetic diversity than endangered and endemic species [21]. However, some studies have shown that rare or endemic species can also have high genetic diversity [22,23]. *C. tsoongiana* exhibited a significantly higher genetic diversity at the species level (*H* = 0.3978) than the average value for perennial herbs (*H* = 0.1240) [24] and many other orchids [12,13,15]. This may be a reflection of the historical pattern of genetic variation in *C. tsoongiana*, that is, a previously wide distribution and only becoming rare recently. In addition, because some distribution areas were influenced by Pleistocene glaciers [25], the current genetic diversity at the species level may be a result of its refuge history. The high diversity in this species may also be related to their perennial long-life habits, which could provide more opportunity to accumulate mutants or special microstructures in different populations due to biotic processes [26].

Genetic diversity within populations is influenced by many factors, such as mating systems, population size, extended time periods with low numbers of individuals, genetic drift and gene flow [7,27]. The mean within-population diversity of *C. tsoongiana* (*H* = 0.1826) was lower than the average value of plant genetic diversity reported by Nybom [28] based on ISSR (*H* = 0.22). This pattern could be attributed to several factors. Firstly, the population sizes of *C. tsoongiana* were small, with only 15 (WN) to 33 (SZ) individuals found, due to habitat destruction and illegal collection. In some cases, plants had become locally extinct in locations recorded in previous literature. Prolonged periods of small population size and population fragmentation can lead to loss in genetic diversity due to the founder effect, genetic bottlenecks and loss of rare alleles [29]. A population with an effective size of 50 is considered the minimum to maintain sufficient genetic variability, while 500 individuals are required to offset effective drift [30]. The relatively high level of genetic diversity within the SZ and TR populations might be because population size reduction occurred very recently in these two locations, especially where that reduction has presented within a generation or two [31]. Secondly, self-incompatibility is very important to maintain high genetic diversity in populations [32]. According to our previous field investigations (unpublished data), however, *C. tsoongiana* is self-compatible. Additionally, decreased genetic diversity can occur due to geitonogamy if flowers on an inflorescence are receptive at the same time, and pollinators frequently interpollinate consecutive flowers on the same inflorescence [7]. Thirdly, a low seed germination rate due to a lack of endosperm and mycorrhizal requirements, low fruit set and vegetative propagation might also lead to relatively low genetic diversity in *C. tsoongiana* within populations [33].

### 3.2. Genetic Differentiation

Differentiation among the locations of *C. tsoongiana* (*G*_st_ = 0.55) populations was higher than the average value (*G*_st_*=* 0.187) obtained from 76 orchid species reviewed by Forrest *et al*. [40]; however, the genetic differentiation values of 71 species (93.4%) in the review were estimated by isozyme analysis, which may not be comparable to values estimated by DNA fragment markers [7,34]. Consequently, it triggered our own summary of levels of genetic differentiation among orchid populations estimated by dominant DNA markers (Table 4). The results showed that the values of *G*_st_ ranged from 0.04 to 0.92, with an average of 0.39. Accordingly, *C. tsoongiana* exhibited relatively high population differentiation compared with other orchids performed by similar markers. This finding supported the low genetic diversity results within populations and the discreet UPGMA clustering of individuals from the same population. The greater similarity of individuals within rather than among the populations indicated that these populations were probably founded by one or a few individuals and that gene flow may be restricted among populations [13]. Gene flow (via pollen and seed) among the *C. tsoongiana* populations (*N*_m_ = 0.4084) was below one, a level of migration that will not prevent continued divergence among populations [47]. Pollen transfer by insects among the populations would be very difficult, due to large geographic distances of more than 150 km. Orchid seeds are usually capable of long-distance dispersal by wind, due to a high content of air and a small size. However, some authors have reported that most orchid seeds fall among maternal plants [48]. Li *et al*. [18] considered that tropical storms or typhoons may assist in seed dispersal, which may explain the over-lapping of individuals from RJ, TR, SZ and WN, as shown by STRUCTURE and UPGMA cluster analysis. Nevertheless, even if seeds are transported over great distances, they are unlikely to encounter the fungal symbiont needed for germination [30]. Therefore, it is likely that geographic isolation, habitat fragmentation, short pollinator movements and limited seed dispersal influence restricted gene flow among the *C. tsoongiana* populations. Genetic drift in continually small population sizes might also play an important role in shaping the present level of population differentiation [34]. The Mantel test indicated that there was no significant relationship between genetic distance and geographic distance, which was further confirmed by the UPGMA dendrogram and PCoA results. This pattern provided further evidence of the existence of genetic drift [49]. Using the STRUCTURE program, the 104 individuals from six *C. tsoongiana* populations were separated into two groups, further confirming that there was no genetic correlation of individuals with their spatial distribution. Similar results were also found in *Liparis loeselii* [5] and *Liparis japonica* [19], with the lack of visible spatial genetic structure in some cases possibly explained by sampled populations having a recent common origin [5].

### 3.3. Implications for Conservation

The ultimate goal of conservation is to ensure the continuous survival of populations and to maintain their evolutionary potential by preserving natural levels of genetic diversity [50]. In a species featuring low gene flow and high genetic differentiation among populations, each population is a special gene pool, and the loss of any such population can cause irreversible loss of genetic diversity [26]. In addition, in small-sized populations, genetic drift might lead to rapid genetic erosion and an increase in extinction risk [11]. Considering that *C. tsoongiana* maintains low within-population genetic diversity and high genetic differentiation among populations and that many of its native habitats and ecosystems have been destroyed by human disturbance, great effort should be made to preserve all extant populations and their habitats to prevent a further decrease in population size and to conserve the overall genetic base of this species. Collection of this orchid should be prohibited. Li and Ge [38] suggested that habitat protection should also protect relevant mycorrhizal fungi and pollinators, which are necessary to continue the lifecycle of orchid species and to maintain and recover their natural populations. A more robust and detailed legal protection policy should be enforced in the local county government, especially for the RJ, SZ and WN populations. Our study indicated an alarming situation for the WN and WYS populations, and pollen or mature seed transfer from other populations via human intervention may be an effective approach to create an artificial gene flow [6]. Transfer of mature plants between populations could also be an effective way to increase the genetic diversity. Furthermore, maintaining a germplasm bank for *ex situ* conservation is also recommended for *C. tsoongiana*. As discussed by Li *et al*. [18], populations (such as SZ and TR) possessing a higher genetic diversity should be given priority in seed banks. Finally, extensive and detailed ecological and biological studies on *C. tsoongiana*, including demographic dynamics, pollination biology, vegetative propagation and identification of fungal associates and germination ecology, should be conducted to achieve comprehensive conservation for this rare endemic orchid [17].

## 4. Materials and Methods

### 4.1. Sample Collection

**Figure 5:**
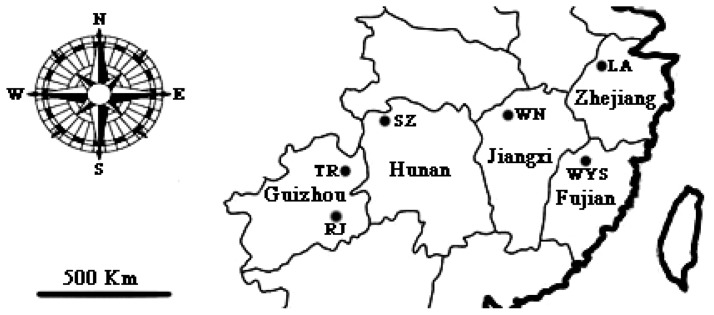
Map of six sampled populations of *C. tsoongiana* in China.

Leaf samples were collected from 104 individuals representing six natural *C. tsoongiana* populations scattered throughout Fujian, Guizhou, Hunan, Jiangxi and Zhejiang provinces in south China (Figure 5). Each population was positioned by GPS, with location details listed in Table 5. Since the plant produces just two or three obovate-lanceolate or oblong leaves, only half of a fresh young blade was harvested from each healthy adult individual to minimize any deleterious effect on their growth. To prevent sampling within clones, leaves were obtained from individual plants located at least 3 m apart. The samples were dried with silica gel in zip-lock plastic bags and later stored at −80 °C in the lab until DNA extraction. Voucher specimens were deposited at the Research Institution of Subtropical Forestry.

### 4.2. DNA Extraction and PCR Amplification

Total genomic DNA was exacted using a plant DNA isolation kit (NEP003-2, Dingguo Biotechnology, Beijing, China), according to the manufacturer’s recommendations. The integrity of DNA samples were assessed through 0.7% agarose gels electrophoresis, and DNA quantification was performed in a Quawell Q5000 UV-Vis spectrophotometer (Quawell, San Jose, CA, USA). The DNA samples were diluted to 20 ng/μL in Tris-EDTA buffer solution and then stored at −20 °C until further analysis.

Initially, 100 ISSR primers (set No. 9, University of British Columbia, Vancouver, Canada) synthesized by Sangon Biotech (Shanghai, China) were screened. Eleven primers (Table 6) that produced reproducible and clear bands and revealed high polymorphism were then selected for further study. The PCR was conducted in 25 μL reaction volumes using optimized conditions as follows: 1× PCR buffer, 3 mM Mg^2+^, 0.3 mM dNTP, 0.4 μM primer, 60 ng of DNA template and 1 U of *Taq* DNA polymerase (Aidlab, Beijing, China). Negative controls, containing all ingredients except the DNA template, were included in each reaction to check the absence of contamination. Amplifications were carried out in a Vapo-protect Gradient thermal cycler (Bio-Rad, Hercules, CA, USA) using the following conditions: 94 °C for 5 min for initial denaturation, 40 cycles of denaturation at 94 °C for 1 min, the appropriate annealing temperature (Table 6) for 1 min, 72 °C extension for 1 min and 72 °C for 10 min as a final extension. The PCR products stained with GelRed (Biotium, Hayward, CA, USA) were electrophoresed on 1.8% (*w/v*) agarose gels in 1× TBE buffer (pH = 8.0) at 100 V for 90 min, with DL2000 ladder (Takara, Shiga, Japan) used as the DNA molecular weight. After running, the gels were visualized and photographed under UV light using a gel analysis system (FR980, Shanghai, China). Each PCR reaction was repeated twice to ensure the reproducibility of the banding pattern.

### 4.3. Data Analysis

The ISSR bands were scored as present (1) or absent (0) for each DNA sample to form a binary matrix. Only fragments showing consistent amplification were considered, while weak and smeared ones were excluded. The following genetic diversity parameters were determined for each population under the assumption that the populations were in Hardy-Weinberg equilibrium using POPGENE version 1.32 [51]: observed number of alleles (*N*_a_); effective number of alleles (*N*_e_); Nei’s gene diversity (*H*); Shannon’s Information index (*I*); percentage of polymorphic loci (*PPL*); total genetic diversity (*H*_t_), genetic diversity within populations (*H*_s_), genetic differentiation among populations (*G*_st_); gene flow (*N*_m_); and Nei’s genetic identity and genetic distance. The same matrix was then used to perform cluster analysis, and an individual-level unweighted pair group was constructed with an arithmetic average (UPGMA) dendrogram using SAHN from NTSYS-pc version 2.20 [52].

Analysis of molecular variance (AMOVA) was used to estimate the variance components and their significance levels of genetic variation within and among the populations using GenAlEx version 6.5 [53] based on 999 permutations. The Φ_pt_ values were also calculated and are considered analogous to *F*_st_, describing the proportion of total variance among populations [54]. Principal coordinate analysis (PCoA) was also employed using the same software to detect the genetic relationships among populations.

A Mantel test for geographic and genetic distances of population pairs was performed to determine if a relationship existed between the two data matrices using TFPGA version 1.3 [55] with 999 permutations.

To infer population structure and estimate genetically homogeneous groups (K) that best fit the data, the Bayesian Method was implemented in STRUCTURE version 2.3.4 [56], assuming the existence of K groups, characterized by a set of allele frequencies for each locus. An admixture model and the allele frequencies-correlated model were adopted, without prior assumptions concerning the population. The number of *K* was set from 1 to 6, and ten independent runs were performed, each with a Markov Chain Monte Carlo (MCMC) of 100,000 repetitions following a burn-in period of 50,000 iterations. Default values were kept for all other parameters. To identify the optimal value of *K*, the STRUCTURE output file was implemented in Structure Harvester version 0.6.93 [57], as per Evanno *et al*. [58].

## 5. Conclusions

Our results indicated that *C. tsoongiana* genetic diversity was high at the species level, but relatively low at the population level. High genetic differentiation was found among populations, which may be attributed to habitat fragmentation, due to anthropopression, geographic isolation, restricted gene flow and genetic drift. Based on these findings, several strategies were proposed for the conservation of this rare orchid.

## Figures and Tables

**Table 1:** Genetic diversity within the populations of *C. tsoongiana*.

Populations	*N*_a_	*N*_e_	*H*	*I*	*PPL*
LA	1.419 (0.496)	1.286 (0.378)	0.163 (0.206)	0.239 (0.296)	41.9%
RJ	1.524 (0.501)	1.314 (0.377)	0.182 (0.200)	0.273 (0.287)	52.4%
SZ	1.726 (0.448)	1.504 (0.389)	0.284 (0.201)	0.415 (0.282)	72.6%
TR	1.694 (0.463)	1.379 (0.360)	0.226 (0.192)	0.342 (0.271)	69.4%
WN	1.307 (0.463)	1.210 (0.356)	0.119 (0.191)	0.174 (0.274)	30.7%
WYS	1.331 (0.472)	1.215 (0.351)	0.122 (0.191)	0.180 (0.275)	33.1%
Average	1.500 (0.474)	1.318 (0.369)	0.183 (0.197)	0.271 (0.281)	50.0%
Species Level	1.968 (0.177)	1.720 (0.285)	0.398 (0.129)	0.576 (0.164)	96.8%

*N*_a_: observed number of alleles; *N*_e_: effective number of alleles; *H*: Nei’s gene diversity; *I*: Shannon’s Information index; *PPL*: the percentage of polymorphic loci; values in brackets are standard deviations.

**Table 2:** Analysis of molecular variance (AMOVA) for the populations of *C. tsoongiana*.

SV	d.f.	SSD	MSD	VC	TVP	Φ _pt_	*p* (rand ≥ data)
Among populations	5	1252.535	250.507	13.878	52%	0.522	0.001
Within populations	98	1246.456	12.719	12.719	48%		
Total	103	2498.991		26.597	100%		

SV: source of variation; d.f.: degree of freedom; SSD: sum of squares; MSD: mean squares; VC: variance component; TVP: total variance percentage; Φ_pt_: the proportion of the total variance among populations.

**Table 3:** Nei’s original measures of genetic identity (above diagonal) and genetic distance (below diagonal) among the populations of *C. tsoongiana*.

Populations	LA	RJ	SZ	TR	WN	WYS
LA	-	0.744	0.699	0.640	0.541	0.796
RJ	0.296	-	0.756	0.757	0.572	0.727
SZ	0.359	0.280	-	0.749	0.670	0.634
TR	0.447	0.279	0.290	-	0.789	0.591
WN	0.615	0.558	0.401	0.237	-	0.528
WYS	0.229	0.319	0.456	0.527	0.638	-

**Table 4:** Genetic differentiation among populations of orchid species based on dominant DNA markers.

Orchid Species	AM	*N*_P_	*N*_I_	*N*_L_	*G*_st_ (*F_st_*)	SR
*Piperia yadonii* (2006)	ISSR	7	210	636	0.42	[7]
*Piperia yadonii* (2007)	ISSR	8	251	622	0.39	[7]
*Cymbidium goeringii*	ISSR	11	325	127	0.22	[11]
*Tipularia discolor*	ISSR	4	56	22	0.42	[12]
*Platanthera aquilonis*	ISSR	7	111	164	0.70	[13]
*Platanthera dilatata*	ISSR	10	152	339	0.49	[13]
*Platanthera huronensis*	ISSR	14	236	517	0.36	[13]
*Gastrodia elata*	ISSR	14	483	77	0.27	[14]
*Dendrobium fimbriatum*	ISSR	5	114	117	0.75	[15]
*Calanthe tsoongiana*	ISSR	6	104	124	0.55	This study
*Zeuxine strateumatica*	RAPD	10	50	71	0.92	[34]
*Eulophia sinensis*	RAPD	7	38	97	0.65	[34]
*Zeuxine gracilis*	RAPD	6	74	77	0.54	[34]
*Goodyera procera*	RAPD	14	343	101	0.39	[35]
*Platanthera leucophaea*	RAPD	10	192	64	0.26	[36]
*Paphiopedilum micranthum*	RAPD	4	161	131	0.20	[37]
*Changnienia amoena*	RAPD	11	216	119	0.43	[38]
*Cattleya labiata*	RAPD and ISSR	7	117	272	0.13	[26]
*Vanda coerulea*	RAPD and ISSR	7	32	226	0.04	[39]
*Liparis loeselii*	AFLP	12	155	108	0.38	[5]
*Dendrobium officinale*	AFLP	12	71	195	0.27	[18]
*Liparis japonica*	AFLP	8	185	406	0.43	[19]
*Spiranthes romanzoffiana*	AFLP	17	205	138	0.89	[40]
*Phragmipedium longifolium*	AFLP	6	160	365	0.20	[41]
*Himantoglossum hircinum*	AFLP	20	211	215	0.15	[42]
*Orchis mascula*	AFLP	15	293	196	0.08	[43]
*Orchis purpurea*	AFLP	9	244	70	0.09	[44]
*Dactylorhiza incarnata*	AFLP	12	250	91	0.35	[45]
*Cypripedium japonicum*	AFLP	6	180	377	0.52	[46]
*Dendrobium loddigesii*	SRAP	7	92	231	0.30	[20]

AM: assessment method; *N*_P_: number of populations sampled; *N*_I_: number of individuals sampled; *N*_L_: number of loci analyzed; SR: source references; RAPD: random amplified polymorphic DNA.

**Table 5:** Sampling details of different populations of *C. tsoongiana* in this study.

Population Code	Locality	Geographical Coordinate	Altitude (m)	Sample Size	Voucher Number
LA	Linan county, Zhejiang	30°21′N,119°25′E	1021	20	J0611
RJ	Rongjiang county, Guizhou	26°23′N,108°12′E	516	20	Q0516
SZ	Sangzhi county, Hunan	29°40′N,110°2′E	363	20	Q0605
TR	Tongren county, Guizhou	27°56′N,180°36′E	818	15	Q0521
WN	Wuning county, Jiangxi	29°6′N,115°17′E	582	9	Q0418
WYS	Wuyishan county, Fujian	27°44′N,117°41′E	705	20	Q0507

**Table 6:** ISSR primers used in the present study.

Primer Name	Sequence (5′→3′)	Annealing Temperature (°C)
UBC813	(CT)_8_T	50
UBC818	(CA)_8_G	52
UBC824	(TC)_8_G	51
UBC828	(TG)_8_A	51
UBC834	(AG)_8_YT	50
UBC843	(CT)_8_RA	50
UBC845	(CT)_8_RG	52
UBC859	(CG)_8_RC	52
UBC868	(GAA)_6_	48
UBC873	(GACA)_4_	49
UBC881	(GGGTG)_3_	56

Y = (C,T); R = (A,G).
